# Supplementary material for: Content-rich biological network constructed by mining PubMed abstracts
Source: BMC Bioinformatics. 2004 Oct 8;5:147. doi: 10.1186/1471-2105-5-147 (PMC528731; doi:10.1186/1471-2105-5-147)
Supplement: Additional File 5 — The original Chilibot query results of the term "long-term potentiation (LTP)" and 22 other terms, limiting the latest references analyzed to the years 1990, 1995, 2000, and 2004. [file 1471-2105-5-147-S5.bz2 › chilibotAdditionalFile5/ltp2004/html/PI-3K_PKA.html]

 


 **PI-3K** and **PKA** 
  
Found 49 abstracts in PubMed,  **30 abstracts were retrieved and analyzed**.  


---

 Search Google  |
 PDF files only 
|  EDU domain only 

---

**Interactive relationship** (e.g. stimulation, inhibition, etc)

- These interactions included a central role of tyrosine phosphorylation for stimulation of  **PKA**  CREB, MAPK and PI3 kinase  [ **PI-3K** ]  PKB, inhibition of  **PKA**  CREB by the MAPK pathway at the level of MAPK kinase 1 or downstream, activation of MAPK signaling by PI3 kinase  [ **PI-3K** ]  and  **PKA**  at the level of extracellular signal regulated kinase 1 2 or upstream, and activation of PKB by MAPK and  **PKA**  signaling at the level of PKB or upstream.  Ref: 12176662 J Endocrinol, 2002

**Parallel relationship** (e.g. studied together, co-existance, homology, etc.)

- These findings are incorporated into a model where the initiation of thyroid cell motility constitutes a morphogenetic checkpoint regulated by coordinated changes in stimulatory ERK, PI3 kinase  [ **PI-3K** ]  and tonic inhibitory cAMP  **PKA**  signaling pathways.  Ref: 11443739 Cell Motil Cytoskeleton, 2001
- In contrast, the stimulatory effect of FGF 1 on ET AR mRNA expression was not altered by PI3 kinase  [ **PI-3K** ] ,  **PKA** , PKC, or adenylate cyclase inhibitors.  Ref: 12851419 J Appl Physiol, 2003
- The effect of ET 1 on glucose uptake was not inhibited by inhibitors for protein kinase C PKC, protein kinase A  **PKA**  and phosphatidylinositol 3 kinase PI3 kinase  [ **PI-3K** ] .  Ref: 11078371 J Cardiovasc Pharmacol, 2000
- We suggest that Bu 2cAMP stimulates Glut 1 translocation to plasma membrane through PI3 kinase  [ **PI-3K** ]  dependent and  **PKA**  independent signaling pathways.  Ref: 11089547 Endocrinology, 2000
- SAPK JNK was dose dependently activated after H2O2 treatment from 10 microM to 1 mM, and a PI 3 kinase  [ **PI-3K** ]  inhibitor wortmaninn, intracellular calcium chelator BAPTA AM, and  **PKA**  activator dibutyl cyclic AMP and forskolin inhibited this activation.  Ref: 11225728 Antioxid Redox SignalAntioxid Redox Signal, 2000
- While inhibitors of the MAPK, PI 3K  [ **PI-3K** ] , and  **PKA**  pathways elicited similar effects on the IGFBP 2 gene promoter activity, irrespective of cellular BTEB1 content, that for JNK had a more pronounced effect on Hec 1 A sublines exhibiting higher BTEB1 expression levels.  Ref: 11953011 DNA Cell Biol, 2002
- Inhibitors of the calcium independent, novel PKC isoforms... PI3 kinase  [ **PI-3K** ] , or  **PKA** , blocked PMA stimulated cIAP 2 mRNA expression, suggesting a role of PKC in PMA mediated cIAP 2 induction.  Ref: 14527959 J Biol Chem, 2003
- In the present study, the use of specific inhibitors confirmed the role of the cyclic AMP  **PKA**  pathway, and also demonstrated a role for the phosphatidylinositol 3 OH kinase PI 3 kinase  [ **PI-3K** ]  neuroprotective pathway in the action of PACAP.  Ref: 14690524 J Neurochem, 2004
- This inhibitory activity of cAMP was not mediated by  **PKA** , but 8 Br cAMP inhibited membrane localization of the p85 regulatory subunit of PI 3 kinase  [ **PI-3K** ] .  Ref: 12939297 Invest Ophthalmol Vis Sci, 2003
- Surprisingly, a different secondary downstream signaling cascade seems to be activated in each estrogen responsive cell population, i.e. phosphatidylinositol 3 kinase PI3 kinase  [ **PI-3K** ]  in GABAergic and cAMP protein kinase A  **PKA**  in dopaminergic neurons, mitogen activated protein kinase MAP kinase in astrocytes.  Ref: 12361721 J Steroid Biochem Mol Biol, 2002
- Therefore, antagonism of beta cell Kv currents by GLP 1 receptor activation requires both cAMP  **PKA**  and PI3 kinase  [ **PI-3K** ]  PKCzeta signaling via trans activation of the EGF receptor.  Ref: 14565957 J Biol Chem, 2003
- Activation of phosphatidylinositol PI 3 kinase  [ **PI-3K** ] , protein kinase A  **PKA**  and protein kinase C PKC is associated with the survival effect elicited by PDGF AB and TGF beta1 against the apoptotic inducer 2 deoxy D ribose dRib in the fat body cell line, IPLB LdFB, from the insect Lymantria dispar.  Ref: 11237421 Cell Biol Int, 2001
- Norepinephrine increases glucose transport in brown adipocytes via beta3 adrenoceptors through a cAMP,  **PKA** , and PI3 kinase  [ **PI-3K** ]  dependent pathway stimulating conventional and novel PKCs.  Ref: 14551227 Endocrinology, 2004
- In addition, activation of  **PKA**  and the phosphoinoside 3 kinase PI 3k  [ **PI-3K** ]  p70 ribosomal S6 kinase cascade was required to have maximal MCP 1 but not IL 8 production.  Ref: 12393171 Cytokine, 2002
- Involvement of PI 3 kinase  [ **PI-3K** ] ,  **PKA**  and PKC in PDGF and TGF beta mediated prevention of 2 deoxy D ribose induced apoptosis in the insect cell line, IPLB LdFB.  Ref: 11237421 Cell Biol Int, 2001
- Likewise, Bu 2cAMP effects on glucose transport and Glut 1 translocation to plasma membrane were repressed by PI3 kinase  [ **PI-3K** ]  inhibitors but not by the protein kinase A  **PKA**  inhibitor H89.  Ref: 11089547 Endocrinology, 2000
- Ethanol exposure accelerates the anti apoptotic effect of PACAP by a mechanism that involves the  **PKA**  and PI 3 kinase  [ **PI-3K** ]  pathways.  Ref: 14690524 J Neurochem, 2004
- Tyrosine kinases, calcium linked pathways, PI 3 kinase  [ **PI-3K** ] ,  **PKA**  and phosphatase inhibition seem to play no or minor roles in the fluoride induced IL 8 response.  Ref: 11567778 Toxicology, 2001
- PI3 kinase  [ **PI-3K** ]  AKT, protein kinase A  **PKA**  and IkappaB kinase appear to be downstream signaling molecules involved in NF kappaB activation as specific inhibitors of these kinases but not inhibitors of ERK MAP kinase or protein kinase C reduced heregulin mediated NF kappaB activation.  Ref: 11960379 Oncogene, 2002
- This enhancement of adenylyl cyclase function was not attenuated by inhibitors of ERK, PKC,  **PKA** , or PI3 kinase  [ **PI-3K** ]  activity but was blunted by inhibition of endogenous p74 raf 1 activity.  Ref: 11327830 Biochemistry, 2001
- To clarify activation mechanisms of stress activated protein kinase C Jun N terminal kinase SAPK JNK during oxidative stress, the roles of phosphatidylinositol 3 kinase PI 3 kinase  [ **PI-3K** ] , concentration of intracellular calcium calcium i, and cyclic AMP dependent kinase  **PKA**  in hydrogen peroxide H2O2 induced SAPK JNK activation were examined in Chinese hamster V79 cells.  Ref: 11225728 Antioxid Redox SignalAntioxid Redox Signal, 2001
